# Comparison of radiovisiography and digital volume tomography to direct surgical measurements in the detection of infrabony defects

**DOI:** 10.4317/jced.50711

**Published:** 2012-02-01

**Authors:** Preeti S. Raichur, Swati B. Setty, Srinath L. Thakur, Venkatesh G. Naikmasur

**Affiliations:** 1 B.D.S. Post Graduate Student. Department of Periodontics and Oral Implantology. S.D.M. College of Dental Sciences, Sattur, Dharwad, India; 2 M.D.S. Professor. Department of Periodontics and Oral Implantology. S.D.M. College of Dental Sciences, Sattur, Dharwad, India; 3 M.D.S., F.D.S.R.C.S. Professor and Head of the department. Department of Periodontics and Oral Implantology. S.D.M. College of Dental Sciences, Sattur, Dharwad, India; 4 M.D.S. Professor and Head of the department. Department of Oral Medicine and Maxillofacial Radiology. S.D.M. College of Dental Sciences, Sattur, Dharwad, India

## Abstract

Objectives: To compare the linear measurements of Radiovisiography (RVG) and Digital volume tomography (DVT) to direct surgical measurements in the detection of periodontal infrabony defects. Study Design: RVG and DVT images were taken prior to periodontal surgery for 28 infrabony periodontal defects. After defect debridement, direct bony defect measurements were made from the cemento enamel junction (CEJ) to the base of the defect (CEJ-BD) and to the alveolar crest (CEJ-AC) with a periodontal probe. These same measurements were made on the RVG and DVT images and then compared to the direct surgical values. Results: DVT correlated strongly with surgical measurements, whereas RVG correlated less favorably (P=0.0109, P=0.0193 respectively).No significant difference for CEJ-AC (P=0.0599) was seen between DVT and surgical measurements; however, there was a significant difference for the distance CEJ-BD (P=0.0105). Conclusion: DVT technique is significantly more accurate than RVG in the detection of infrabony periodontal defects.

** Key words:**Tomography, volume computed, dental radiovisiography, periodontal diseases.

## Introduction

The diagnosis of periodontal diseases involves careful analysis of the case history and evaluation of the clinical signs and symptoms, as well as the results of various tests like probing, mobility assessment, radiographs, blood tests and biopsies. Dental radiography is a non invasive diagnostic technique primarily used to survey morphological and pathological changes of diagnostic interest in the teeth and jaws. This is the technology that dental practitioners are most familiar and comfortable working with, in terms of technique and interpretation.

The radiographic techniques routinely used in periodontal diagnostics include panoramic, bitewing and periap-ical radiography (1). These radiographs are an adjunct to clinical examination and represent a valuable aid in the diagnosis of periodontal diseases, determination of prognosis and the assessment of treatment outcomes. Radiographs also serve as a reference image to evaluate the progress of cases over time. They reveal the pattern, extent of interdental and interradicular bone resorption, root length, periodontal ligament space and changes in the periapical region. But one of the major disadvantages with this technique is the projection of alveolar bone on a 2-dimensional (2-D) plane where many anatomical structures may overly lesions in the trabecular bone (2). Limited differentiation between the buccal and lingual alveolar bone also makes the topography and extent of bone lesions or dehiscences impossible to evaluate with certainty.

Rapid development of computer technology has opened the way to digital and advanced imaging modalities where high performance processors manipulate and handle large amounts of data. Since the early 1980s, Com-puted Tomography (CT) has been used in dental diagnostics, depicting the osseous structures in 3 planes, true to scale. (3) But its use in a dental set up has a few disadvantages, namely the cost and bulk of the equipment as well as the increased effective radiation dose to the patients.

Digital Volume Tomography (DVT) has been in use in dentistry since the late 1990s as a 3-dimensional radiographic technique (4). Comparatively low radiation exposure particularly when just a small volume is examined seems to be a major advantage with this technique. DVT or Cone Beam Volumetric Tomography (5) (CBCT) provides high quality thin slice images. It emits an x-ray beam shaped like a cone, covering the entire region of interest and also comprises of a high performance panel detector. Serial cross sectional views can be made in the axial, sagittal, and coronal planes. In addition, tomographic sections in a curved plane corresponding to the dental arch can be made in DVT which is not possible with the CT. These unique features make the technique not only invaluable in diagnosing deep infrabony periodontal defects but also in measuring the exact depth of the defect as well. It also reveals deep circumferential defects which could make periodontal regenerative therapy impractical, thus eliciting a hopeless tooth prognosis.

Previous studies have been conducted using cadaveric or animal models in the detection of infra bony defects comparing routine RVG to DVT. But studies in humans are scarce. Thus the study aimed to compare the linear measurements of infrabony periodontal defects obtained by Radiovisiography (RVG), Digital volume tomography (DVT) and direct surgical measurements (SUR).

## Material and Methods

The study was conducted in the Department of Periodontics and the Department of Oral and Maxillofacial Radiology, SDMCDS, Dharwad, India. 7 patients (3 males and 4 females) with moderate to severe chronic periodontitis who were scheduled for periodontal surgery took part in the present study. Their age ranged from 30 to 60 years, with a mean age of 45 years. They were informed about the study protocol, risks and benefits of the diagnostic and therapeutic procedures and consent was obtained. Ethical clearance from the local ethical committee was obtained. A sample size of 28 sites was taken for the assessment. Each defect was the unit of analysis.

Radiographic examination 

RVG: After completion of initial periodontal treatment which included scaling, root planning and oral hygiene instructions, standardized intra oral periapical radiographs were taken incorporating paralleling technique for the teeth exhibiting vertical interproximal bone loss (infra bony defects). The RVG sensor was positioned in the mouth parallel to the long axis of the desired tooth. The X-ray tubehead was aimed at right angles (vertically and horizontally) to both the tooth and the sensor. A paralleling device† was used for this purpose. Images were obtained with a size #2 charged couple device (CCD) intraoral digital sensor§ and a standard X-ray unit* operating at 60-63 kV, 8 mA and 0.25-0.32 sec. A rectangular collimator was used with a Focal-Film distance of 30 cm. The exposure time ranged between 0.32-0.5 sec. based on the tooth type.

†-Planmeca Dixi V4 Sensor Holder, Size 2, Helsinki, Finland.

§-Planmeca Dixi®

*-Planmeca Prostyle Intra, Helsinki, Finland.

#-XCP, RINN Corp Film Holding System

DVT: Limited volume (50x37 mm) 3-Dimensional images of the required site were obtained with a digital imaging system¤. The patient’s mid sagittal plane was centered. Occlusal plane was positioned horizontally to the scan plane. Exposure parameters were set at 70-74 kV, 10 mA and 10.8 seconds.

Assessment of bone height:

The following measurements in RVG were made using inbuilt software on a 15 inch, 1,680 ×1,050 pixel resolution LCD computer screen under ambient viewing conditions. Cemento-enamel junction to alveolar crest (CEJ-AC) and Cemento-enamel junction to base of the defect (CEJ-BD) were measured.

 For the measurements in DVT, a 19 inch LCD monitor? with 1280 x 1024 pixel resolution, 800:1 contrast ratio was used. A bone window was used to obtain the best bone contrast. A dental imaging software? was used for this purpose. The data of DVT images were sliced in three dimensions. Oblique reconstruction was used to reorient the volume so that the interproximal surface of the tooth could be positioned for measurements. The slice thickness could be varied from 0.2 mm to as large as 6.0 mm. But 1.0mm slices were used in the present investigation because this increment closely represents the same error of measurement as when using a periodontal probe; also smaller slices decreased the image resolution. The three axes (X, Y, and Z) of the DVT images were sequentially analyzed to locate the most apical point of the BD or the most coronal aspect of AC. Each slice was analyzed for the separate measures, because the extreme points for BD and AC were usually located in two different slices.

¤-Kodak 9000 C3D 

?-HP L 1910, Hewlett-Packard, USA.

?-Kodak Dental Imaging Software (Windows edition 6.8 Carestream Health Inc.NY USA).

For the surgical procedure, full thickness mucoperiosteal flaps were reflected under local anaesthesia and the defects were debrided. Direct surgical hard tissue measurements were made using a UNC 15 probe. Cemento enamel junction or the base of an existing restoration was used as the reference point. Measurements included CEJ-AC and CEJ-BD. Alveolar crest and base of the defect were determined from the buccal/labial and lin-gual/palatal aspects of each defect. Smallest CEJ-AC value, largest CEJ-BD values were used.

## Statistical Analysis

To determine the actual sample size, a pilot study was conducted, based on the results of the pilot study, a total of 28 defects or sites were included under 5% alpha error and 83% of power of the test to detect the real significant difference. The one way ANOVA was used to compare the three techniques (RVG, DVT and SUR) with CEJ-AC and CEJ-BD measurements followed by Newman Keuls multiple post hoc procedures used for pair wise comparison of the three methods. A statistical significance was set at 5% level of significance (p<0.05).

## Results

 Table 1, 28 infrabony defects in 7 patients provided data for analysis. ANOVA was done to determine if there was any statistically significant difference between the three investigative modalities (RVG, DVT, SUR) for CEJ-AC and CEJ-BD measurements. No differences were seen in the mean measurements between RVG, DVT, SUR techniques (P=0.0599) in the CEJ-AC group whereas the same measurements showed a significant differ-ence (P=0.0105) in the CEJ-BD group. When pairwise comparison was done by Newman Keuls multiple com-parison post hoc procedure of the three methods, there was a significant difference seen between the RVG vs. DVT as well as the direct surgical measurements (P=0.0109, P=0.0193 respectively). No significant differences were observed between DVT and direct surgical measurements.

**Table 1:**
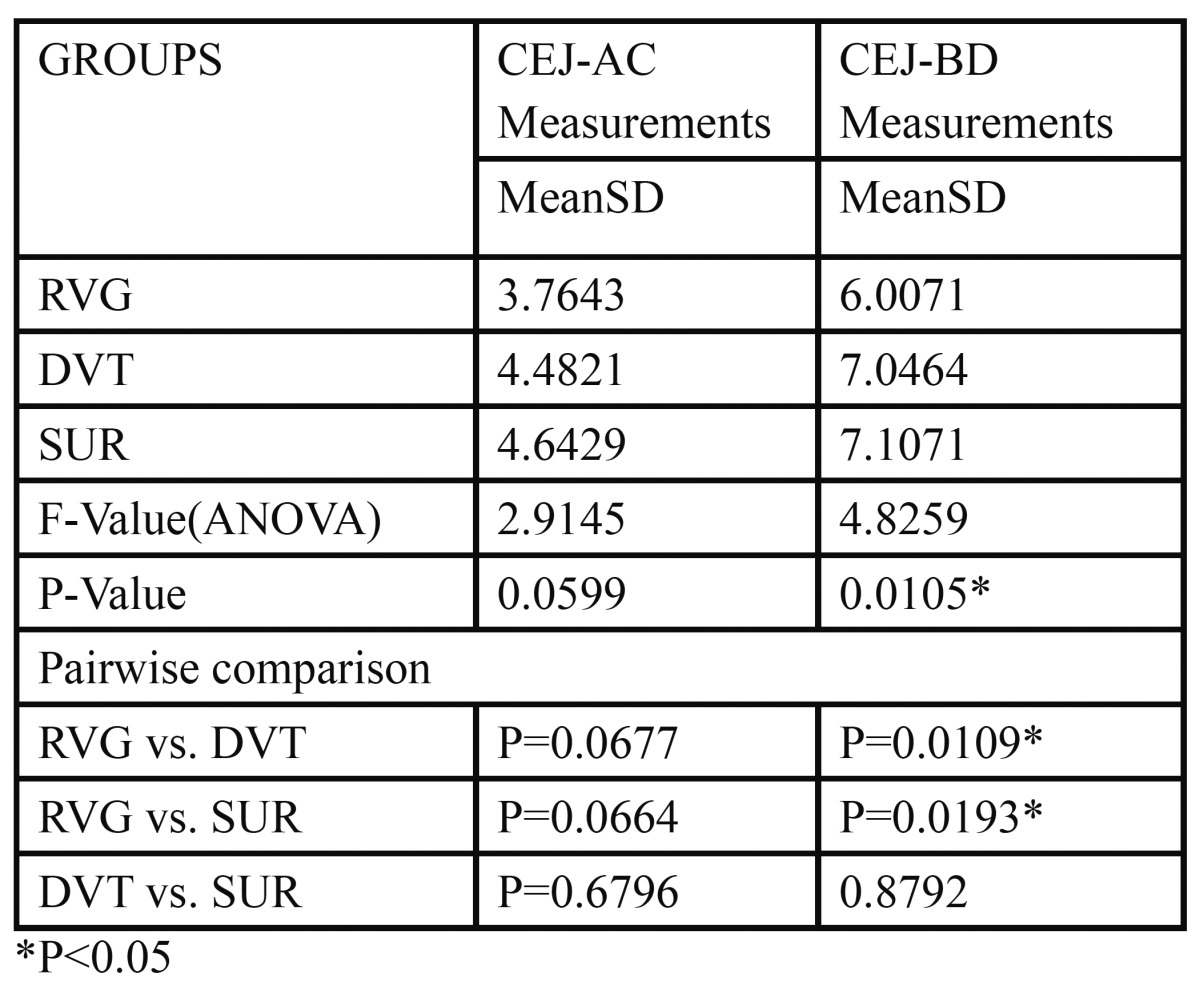
Comparison of the three techniques with CEJ-AC and CEJ-BD measurements.

## Discussion

This study compared the direct surgical dimensions of infrabony periodontal defects to measurements made on RVG and DVT images for diagnostic purposes. Surgical measurements were considered the “gold standard” values against which the radiographic measurements were compared. Only two dimensions of the supporting alveolar bone are discernible on an RVG whereas DVT in addition allows assessment in all three dimensions and lets the viewer locate the deepest portion of the defect, wherever it may be along the tooth surface. The technique of RVG is simple, and measurements can be performed easily using a number of digital measuring tools. It is the technology that practitioners are most familiar and comfortable with, in terms of technique and interpretation. Wolf et al. assessed the reproducibility and validity of linear measurements of interproximal bone loss in infrabony defects on digitized radiographic images after application of different filters and magnifications (6). The digital manipulations of radiographic images increased neither the reproducibility as assessed by repeated measurements nor the validity as referred to surgical measurements of the radiographic assessment of alveolar bone loss. We therefore in our present study did not use any filters or magnification.

Eickholz et al. examined the accuracy of linear measurements on radiographs of interproximal bone loss in infrabony defects utilizing the gold standard of surgical measurements (7). In all radiographs, the linear distance from CEJ to BD was assessed. Radiographic and surgical assessments were compared. The radiographic assessments underestimated bone loss as compared to surgical measurements. These results were in accordance with our study.

Correlations between DVT and direct surgical measurements were higher than those between RVG and surgical measurements. DVT measurements were found to be within 1 or 2mm of surgical measurements than RVG measurements. This suggests that data gathered from DVT images more accurately reflect bony defect dimensions than do data from RVG images. Artificial osseous defects were created on mandibles of dry skulls and were subjected to CBCT scanning, periapical radiography (PA) and direct measurements using a periodontal probe by Misch et al. These were compared to an electronic caliper that was used as a standard reference. All the three techniques showed similar efficacy in identifying interproximal periodontal defects (8). The results of the present study differed. This might have been possible because geometric alignment of intraoral radiographs on dry skulls is easier, thus providing better images than can be achieved in a living human. Extrapolating these results to living humans seems somewhat farfetched.

DVT has been applied in vivo for the assessment of furcation invasion and treatment planning in maxillary molars (9). In a study of native pig and human mandibles, Mengel et al. investigated the accuracy and quality of the representation of periodontal defects by intraoral radiography (IR), panoramic radiography (PR), CT and DVT in comparison with histologic specimens. CT and DVT were far more superior compared to IR and PR. There was a negligible variation between CT, DVT and histologic specimens in measuring the extent of the periodontal defects (10). This suggests that CT and DVT are accurate measurement tools.

Grimard et al. compared the measurements from digital IR and DVT images to direct surgical measurements for the evaluation of treatment outcomes, specifically increase in alveolar bone height after regenerative procedures (11). No significant difference was seen for the distance from CEJ-AC for measurement before surgery and for reentry; however, there was a significant difference for the distance from the CEJ-BD, with DVT measurements underestimating the surgical measurements by 0.5 – 1.1mm for reentry and 0.9 – 0.8mm for the initial measurement. Our study also showed a difference between surgical measurements and DVT for evaluating CEJ to BD but not for CEJ to AC. The DVT measurement technique underestimated surgical measurements of CEJ to BD, whereas there was minimal difference between DVT and surgical measurements for CEJ to AC. DVT image was acquired before the scheduled surgery. Thorough debridement of the osseous defect might have removed some bone at the base of the defect, resulting in deeper CEJ to BD measurements as compared to the DVT taken prior to surgery. The alveolar crest is more cortical in nature and less prone to bone removal during defect debridement. Thus fewer variations were seen from direct surgical measurements than DVT or RVG measurements. Also, probe angulation may be greater when assessing the base of the defect compared to assessing the alveolar crest because the base of the interproximal defect often lies directly under the contact area (12). Radiation dose is always a concern for using conventional CT. However, the radiation dose of DVT is reported to be upto 15 times less than conventional CT. Comparing skin exposure of traditional CT versus a small-view CBCT, Honda et al. reported a total radiation reduction from 160 to 1.19 mSv (13). The DVT images emit a similar effective dose of radiation (7.4 µSv) to the patient as that of a panoramic radiograph (160 µSv with conventional CT) (14). Finally, it is likely that radiation exposure will further decrease as technology evolves. A diagnostic system that combines and superimposes the radiographic image with probing data or biologic information, thereby providing a comprehensive periodontal chart and equipment with still lesser radiation exposure need to be developed.

## Conclusions

This study showed that DVT technique was significantly more accurate than RVG in the detection of infrabony periodontal defects. Also, it may serve in the detection of furcation involvements, fenestrations and dehiscences. DVT may be invaluable in regenerative procedures, obviating the need for reentry procedures.
